# Artificial Intelligence in Digital Pathology to Advance Cancer Immunotherapy

**Published:** 2022-05-25

**Authors:** Pingjun Chen, Jianjun Zhang, Jia Wu

**Affiliations:** 1 Departments of Imaging Physics, The University of Texas MD Anderson Cancer Center, Houston, TX, USA; 2 Thoracic/Head and Neck Medical Oncology, The University of Texas MD Anderson Cancer Center, Houston, TX, USA; 3 Genomic Medicine, The University of Texas MD Anderson Cancer Center, Houston, TX, USA

**Keywords:** Artificial intelligence, Digital pathology, Immunotherapy, Multiplex bioimaging

## Abstract

Immune-checkpoint inhibitors (ICIs) have revolutionized the treatment of many malignancies. For instance, in lung cancer, however, only 20^~^30% of patients can achieve durable clinical benefits from ICI monotherapy. Histopathologic and molecular features such as histological type, PD-L1 expression, and tumor mutation burden (TMB), play a paramount role in selecting appropriate regimens for cancer treatment in the era of immunotherapy. Unfortunately, none of the existing features are exclusive predictive biomarkers. Thus, there is an imperative need to pinpoint more effective biomarkers to identify patients who may achieve the most benefit from ICIs. The adoption of digital pathology in clinical flow, as being powered by artificial intelligence (AI) especially deep learning, has catalyzed the automated analysis of tissue slides. With the breakthrough of multiplex bioimaging technology, researchers can comprehensively characterize the tumor microenvironment, including the different immune cells’ distribution, function, and interaction. Here, we briefly summarize recent AI studies in digital pathology and share our perspective on emerging paradigms and directions to advance the development of immunotherapy biomarkers.

## Introduction

Immunotherapy by immune-checkpoint inhibitors (ICIs) is a revolutionary cancer treatment that harnesses the body’s immune system to fight against cancer cells, which has transformed the landscape of cancer treatment [1]. Histopathologic and molecular features such as histological type, PD-L1 expression, and tumor mutation burden (TMB), play a paramount role in selecting appropriate regimens for cancer treatment in the era of immunotherapy. However, the clinical efficacy of immunotherapy varies significantly among different cancer types and across individual patients. The overall response rates to ICI monotherapy are low with only 20~30% observed in lung cancer [1]. Therefore, there is an urgent need to identify effective biomarkers to select patients most likely to benefit particularly for metastatic tumors, where the biopsy specimens are often scarce and not amendable for multi-omics profiling.

With the rapid development of hardware and software for whole side imaging (WSI), particularly since the Food and Drug Administration (FDA) approved the WSI system to be used for primary diagnosis in 2017 [2], digital pathology has gained substantial momentum in scientific research and started to propagate to tackle many clinical challenges. Compared to conventional pathology flow, digital pathology is more flexible and efficient in managing stained sections, sharing images for external consultations, and training next-generation pathologists. Most importantly, computer-aided diagnosis (CAD) can be embedded into the pathology flow powered by the digital pathology framework [3]. Artificial intelligence (AI) has achieved revolutionary breakthroughs in computer vision, gaming, and medicine, among others. Deep learning, especially convolutional neural networks (CNNs), has boosted the state-of-the-art performance in most biomedical image analysis tasks, including pathology image analysis [4]. In a survey of 487 pathologists across 54 countries, nearly 80% of respondents expected the integration of AI into diagnostic pathology practice within the next decade [5]. In addition, the great synergy between digital pathology and AI provides unprecedented opportunities to advance cancer diagnosis and therapeutics, especially immunotherapy. In this short review, we will first review some representative AI applications in digital pathology, and then discuss the emerging paradigms and directions in identifying biomarkers to improve immunotherapy response and spare patients from adverse events.

### Machine Learning Algorithms to Phenotyping Tumor Immune Microenvironment

Routine Hematoxylin and Eosin Staining (H&E): H&E is commonly used to reveal the nuclei morphology, and from which tumor-infiltrating lymphocytes (TILs) have demonstrated strong clinical values related to immunotherapy in various tumor types. There are ongoing efforts to automate TILs scoring from H&E slides using various CNN models [6], which can potentially overcome the tedious counting to accelerate its clinical translation. In addition, tertiary lymphoid structure (TLS), as an ectopic lymphoid formation that can fuel and sustain the immune response, is another emerging biomarker for immunotherapy [7], where pilot studies showed the feasibility of directly index TLS with CNN models [8]. Furthermore, advanced machine learning models are being developed on H&E scans to infer the immunotherapy-related metrics from other platforms, such as microsatellite instability (MSI) [9], and tumor mutational burden (TMB) [10], programmed death-ligand 1 (PD-L1) status and more [11].

Immunohistochemistry (IHC) Staining: PD-L1, as one of the most studied biomarkers, is clinically used to stratify immune-checkpoint inhibitors (ICIs) based therapies in lung and other cancer types [12]. There are pilot studies that developed AI-enabled automatic PD-L1 scoring systems based on different assays in NSCLC [13], breast cancer [14], and head and neck cancer [15]. For instance, Wang X, et al. (2021) proposed a deep learning-based artificial intelligence-assisted (AI-assisted) model to score PD-L1 expression of tumor-infiltrating immune cells (IC) in breast cancer, and they demonstrated the proposed AI-assisted scoring can improve PD-L1 assay (SP-142) assessment on both accuracy and concordance via a large multi-institutional ring study on a total of 109 PD-L1 IHC stained images [14]. Besides, there is active research on other biomarkers (e.g., CD8) for immunotherapy response assessment. For example, Boquet I, et al. (2022) compared the automated Immunoscore based on AI-assisted digital pathology with four pathologists’ visual scoring and manifested the superiority of automatic Immunoscore measurement on both the performance and consistency [16]. Moreover, the CD8xPD-L1 signature obtained by automated NSCLC biopsy image analysis of provided better stratification for patients who received durvalumab, and thus potentially able to identify NSCLC patients who effectively respond to durvalumab treatment [17]. However, the current developed IHC scoring system is mainly focused on the ratio of one or two particular biomarkers, while ignoring tumors’ spatial heterogeneity within a tumor, which may also play a role in driving the response to immunotherapy [12].

**Figure 1:**
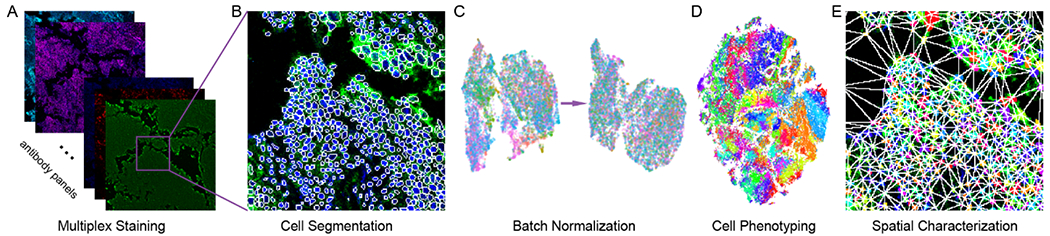
Key components in multiplex bioimaging analysis framework.

Next Generation Multiplex Bioimaging: Multiplex techniques can simultaneously profile dozens of proteins in situ, enabling the systematic study of cellular compositions, functions, and interactions [11]. The multiplex imaging with big data imposes a challenge for pathologists to directly evaluate these slides, where AI-powered computational tools are being developed to characterize the tumor microenvironment [11]. The AI framework contains several key steps (Figure 1): cell segmentation, batch normalization, cell phenotyping, and spatial characterization. The most challenging task lies in accurately recognizing the precise phenotypes of these cells. The unsupervised clustering algorithms (e.g., FlowSOM [18]) are widely for cell phenotyping, as a replacement for manual gating. However, the unsupervised algorithm doesn’t mean fully automated or unbiased interrogation, since human involvement is still required for choosing the hyperparameters behind these clustering algorithms, and most importantly, refining and confirming these cluster phenotypes using biology knowledge. Cell phenotyping is still a semisupervised, iterative computational process guided by domain expert interpretation [19]. Genuine unbiased approaches for multiple imaging cell phenotyping are in urgent need to facilitate the large-scale tumor microenvironment study.

In addition, multiplex imaging and analytics systems have been used to interrogate biological underpinnings for breast cancer progression from the pre-invasive stage [20] and the antitumoral immunity at the invasive front of colorectal cancer [21].

## Future Outlook

### Multimodal Data Integration:

**Figure 2:**
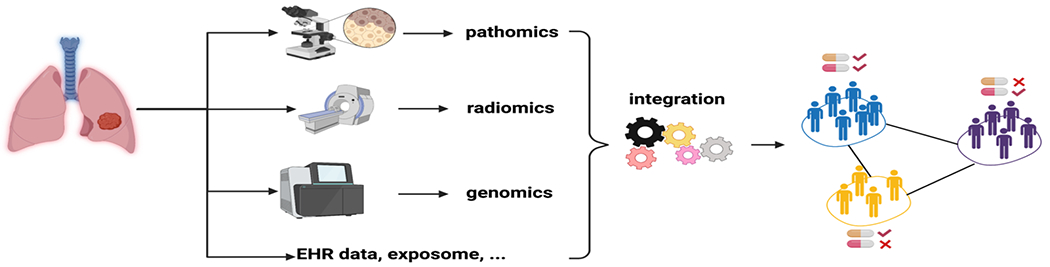
Integration of rich patient data from various platforms for precise treatment stratification.

The information from a single platform or modality will carries limited information to characterize the patients. It will be a fruitful direction to combine the H&E with multiplex imaging to better profile TME. Moreover, such integration can be extended to various medical data, including radiomics, genomics, and electronic health records clinical data [22] (Figure 2). We envision that integrating different resources will offer a holistic view of patient status and enable precision immunotherapy [22]. Meanwhile, harnessing data from different platforms, especially based on unstructured data sources including raw radiology and histology scans, will require sophisticated and robust computational infrastructures. On the other hand, correlating the histology features with molecular assays will allow us to start pinpointing the dysregulated pathways that potentially drive the abnormality as manifested on histology slides [22].

### New Histopathology Subtype Discovery:

It is well known that cancer is not just one disease but a group of distinct molecular or pathological subtypes. Given the rich immunogenomic data, six immune subtypes were identified across 33 cancer types [23]. Similarly, four radiomics subtypes were identified in three malignancies, with one subtype associated with improved survival after immunotherapy in lung cancer [24]. In chronic lymphocytic leukemia, three cell types (CLL-like, aCLL-like, and RT-like) were recognized via unsupervised clustering. Based on these newly discovered cell types, extracted features presented the most robust diagnostic performance [25]. Hence, we envision that given the comprehensive AI-derived profiling, new histopathological subtypes will be discovered to advance our understanding of tumor heterogeneity at the cellular level.

## Conclusion

Innovations in digital pathology technology and AI have presented an unprecedented opportunity to advance biomarker development in immunotherapy. Though these approaches have demonstrated promising results, AI in digital pathology is still in its infancy. Data security and privacy put additional challenges to training and validating robust biomarkers in a multicenter setting of heterogeneous protocols. Powered by emerging federated learning [26], it becomes feasible to bring these models to the siloed data and train them securely. In the next few years, there are several hurdles that the field needs to overcome before we can start incorporating them into the clinical workflow, including prospective testing, FDA approval, and pathologist and oncologist buy-in. On the other hand, current AI applications are tested in a well-controlled environment, where these algorithms are usually developed to target specific challenges – so-called “narrow AI”. In the future, breakthroughs are critical to building next-generation AI tools to handle complicated pathology challenges. Though the field may tend to overestimate what AI can achieve in the short term, we are optimistic that AI in digital pathology will significantly impact the landscape of immunotherapy selection in the coming years.
